# A Hand Gesture Recognition Strategy Based on Virtual-Dimension Increase of EMG

**DOI:** 10.34133/cbsystems.0066

**Published:** 2024-01-29

**Authors:** Yuxuan Wang, Ye Tian, Jinying Zhu, Haotian She, Yinlai Jiang, Zhihong Jiang, Hiroshi Yokoi

**Affiliations:** ^1^ School of Mechatronical Engineering, Beijing Institute of Technology, Beijing, China.; ^2^ Faculty of Informatics and Engineering, The University of Electro-Communications, Tokyo, Japan.

## Abstract

The electromyography(EMG) signal is the biocurrent associated with muscle contraction and can be used as the input signal to a myoelectric intelligent bionic hand to control different gestures of the hand. Increasing the number of myoelectric-signal channels can yield richer information of motion intention and improve the accuracy of gesture recognition. However, as the number of acquisition channels increases, its effect on the improvement of the accuracy of gesture recognition gradually diminishes, resulting in the improvement of the control effect reaching a plateau. To address these problems, this paper presents a proposed method to improve gesture recognition accuracy by virtually increasing the number of EMG signal channels. This method is able to improve the recognition accuracy of various gestures by virtually increasing the number of EMG signal channels and enriching the motion intention information extracted from data collected from a certain number of physical channels, ultimately providing a solution to the issue of the recognition accuracy plateau caused by saturation of information from physical recordings. Meanwhile, based on the idea of the filtered feature selection method, a quantitative measure of sample sets (separability of feature vectors [SFV]) derived from the divergence and correlation of the extracted features is introduced. The SFV value can predict the classification effect before performing the classification, and the effectiveness of the virtual-dimension increase strategy is verified from the perspective of feature set differentiability change. Compared to the statistical motion intention recognition success rate, SFV is a more representative and faster measure of classification effectiveness and is also suitable for small sample sets.

## Introduction

The hand plays an irreplaceable role in executing routine tasks, such as writing, using cutlery, picking up items, and driving. However, due to car accidents, mining accidents, diseases, and so on, the number of amputees is rising year by year. The lack of a limb can have a important impact on an individual’s life, work, and psychological well-being. With the continuous development of science and technology, wearing intelligent bionic prostheses has gradually become an important way to rebuild the function of limbs and improve the living standards of people who have undergone amputations.

Currently, the field of intelligent bionic prostheses is developing rapidly, and many research institutions, domestically and abroad, have developed many relevant products, such as the Hannes Bionic Hand that was introduced by the Italian Institute of Technology and the Italian INAIL Prosthetic Center in 2020 [1]. The Hannes Bionic Hand is very similar to a human hand and can help amputees regain most hand function; it has a differential underactuated mechanism and weighs only approximately 480 g. Gu et al. [2] designed a pneumatic prosthetic hand in 2021, which has 6 active degrees of freedom and can provide haptic feedback through electrical stimulation; its flexibility is better than that of conventional prosthetic hands. The mainstream intelligent bionic prostheses on the market have their own advantages, and most of them have multiple degrees of freedom and are highly bionic, allowing them to imitate most of the gestures used in daily life and making them convenient for amputees.

Human surface electromyography(EMG) signals are widely used for motion control of intelligent bionic prostheses due to their easy acquisition and rich information content [3]. Usually, the accuracy of motion control is positively correlated with the amount of useful information that can be collected. Given this principle, researchers often increase the amount of valid information obtained by adding acquisition electrodes or sensors. Jiang et al. [4] incorporated an inertial measurement unit into the signal acquisition system to enable the development of a wristband with good real-time gesture recognition. Thalmic Labs in Canada introduced the MYO arm ring, which has 8 acquisition channels and Bluetooth transmission, but the device does not allow for flexible adjustment of the signal acquisition position because its 8 acquisition electrodes are connected [5]. In the United States, Delsys introduced a portable high-density EMG signal acquisition device called Trigno, which can use up to 128 acquisition channels for EMG signal acquisition [6]. In Italy, OT Bioelettronica developed the Quattrocento, a desktop acquisition device that can acquire signals from up to 384 acquisition channels simultaneously [7]. In the Netherlands, TMSi developed the SAGA system, which has a resolution of 24 bits and can acquire information from 64 acquisition channels simultaneously [8]. By increasing the number of acquisition channels, richer information about the motion intent can be extracted. However, increasing the numbers of channels also increases the complexity, weight, and cost of EMG signal acquisition devices. Furthermore, the large number of acquisition channels makes it difficult to achieve daily portability, limiting the transition of these methods from the laboratory to practical applications.

The method of improving the accuracy of gesture recognition by increasing the number of signal acquisition channels will not only lead to a decrease in the portability of the dummy hand and more interference factors in the acquisition process, but also with the increase in the number of acquisition channels, its effect on the improvement of the success rate of gesture recognition gradually diminishes, resulting in the improvement of the control effect reaching a plateau. To improve control with fewer acquisition channels, He et al. [9] demonstrated that through spatial filtering and electrode position optimization, the myoelectric control performance with only 2 surface electromyography electrodes was similar to that with 4 electrodes. Krasoulis et al. [10] significantly improved classification performance by combining surface electromyography with inertial measurement. Naik et al. [11] proposed an electromyographic control technique based on independent component analysis and Icasso clustering, which utilizes a combination of source separation and Icasso clustering to improve the classification performance of independent finger movements for transradial amputee subjects and ultimately achieves higher classification accuracy. However, the human hand needs the synergistic movement of several muscle groups to complete any action, and the method of acquiring signals from randomized individual muscle groups through only physical electrodes ignores the synergistic nature of arm muscle contractions when the hand performs an action, so existing methods are not possible to conduct targeted signal collection and information fusion between channels based on the participation of various muscles in the movement. This results in the inability to fully utilize the differences between key muscles in the gesture recognition process to improve recognition performance.

Meanwhile, the gesture recognition success rate is commonly used by researchers to measure the classification effect [12,13]. However, the results of this method need to be reflected after the features are classified and recognized, and it does not reflect the intrinsic divisibility of the feature set. To address this problem, He et al. [14] used 3 indices based on the Mahalanobis distance in their experiment to quantify the differences between different algorithms in the feature space with and without electrode movement, thus providing a more intuitive representation of the changes in the feature set. Based on the Euclidean distance between points and points in space, the separability of feature vectors (SFV) metric is introduced in this paper. It can effectively predict the classification effect by calculating the ratio of intraclass dispersion to interclass dispersion of the feature set and at the same time can quantify the divisibility of the feature set.

## Materials and Methods

### Intelligent bionic prosthetic-hand design

**Fig. 1.**
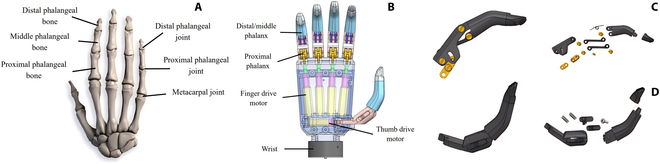
Structure of the human hand and the prosthetic hand. (A) Diagram of bone structure of the human hand. (B) Overall structure of prosthetic hand. (C) Structure of the index finger and its exploded view. (D) Thumb structure and its exploded view.

The human hand is an extremely complex motor system that allows humans to manipulate tools flexibly and perform numerous actions [15,16]. The skeletal structure of the human hand is shown in the Fig 1A. Design of prosthetic-hand structures based on the bone structure of healthy adults can optimize the patient’s experience of using the prosthesis.

The structure and shape of the myoelectric prosthetic hand designed in this paper is very similar to that of a human hand, with 5 fingers that are connected to the palm portion of the hand, and each finger has 2 degrees of freedom and is driven by a separate motor. The design dimensions of each finger and the rotation angle of each joint are shown in Table 1. The motor and the control circuit are placed in the palm to enhance the integration and dexterity of the prosthetic hand, as shown in Fig 1B. The 4 fingers of the prosthetic hand, namely, the index, middle, ring, and little fingers, are similar in structure. The exploded structure diagram of the index finger are shown in Fig 1C. The thumb of the prosthetic hand has 2 knuckles and can perform inward and outward movements. Its exploded view are shown in Fig. 1D.

**Table 1.** Design dimensions of each finger and rotation angle of each joint

Fingers	Proximal phalanx length/mm	Middle phalange length/mm	Distal phalangeal length (mm)	Maximum angle of proximal finger joint (°)	Maximum angle of middle/distal phalanx (°)
Thumb	30	20	38	90	90
Index finger	35	20	27	70	115
Middle finger	35	23	33	70	115
Ring finger	35	20	27	70	115
Little finger	32	15	19	70	115

### Gesture intention recognition strategy based on virtual-dimension increase

#### Virtual-dimension increase

The pattern recognition method based on the time and frequency domain feature extraction of the EMG signal for controlling a prosthetic hand has a high recognition accuracy for different gestures with a large number of data collection electrodes, which can basically meet the requirements for daily use. The most direct and effective method of improving the accuracy of gesture recognition is to increase the number of electrodes that extract the electromyographic signal features of each muscle group of the arm when the hand is moving to provide more gesture information for gesture recognition. However, while this approach increases the accuracy of gesture recognition, it also increases the complexity of hardware devices and built-in algorithms and the dependence of gesture recognition on acquisition channels. If the user does not have enough muscle groups to support signal acquisition (e.g., incomplete limbs) or enough valid signals cannot be acquired, the recognition success rate will be significantly reduced. At the same time, hand movements are accomplished by multiple muscle groups acting in concert, so it is possible to obtain the implicit information carried across different channels without adding additional electrodes by analyzing the differences between different muscle groups in the arm during movement.

In multichannel EMG signals, the EMG data acquired from different channels can be processed and fused. There are 3 main levels of fusion, namely, data-level fusion, feature-level fusion, and decision-level fusion. Data-level fusion is performed after the acquisition of the raw data, which are analyzed and processed, and the fused data are used for the subsequent steps. Feature-level fusion entails analyzing the original signal to obtain the required feature information and to provide a reliable source of information for subsequent decision-making. Fusion at the decision level first requires basic processing such as feature extraction, then preliminary analysis of the target, and finally fusion. In this paper, the data-level fusion is chosen for processing EMG signals from different channels, with the aim of obtaining additional information that exists between EMG signals from different muscle groups. In this section, the addition of virtual dimensions to existing EMG signals is proposed. The term “virtual” refers to the use of data fusion to add new information without using additional physical channels. Each physical channel acquires the electrical signal reflecting the activity of one muscle, representing one dimension of data information. The term “dimensioning” refers to the processing of data across 2 or more different channels. This information can be merged with that of the original EMG signal as a new channel dimension and can be used to improve the accuracy of gesture intention recognition.

It is first necessary to process the EMG data acquired from each channel, intercepting 200 ms of data for processing and analysis of the feature values that can represent the muscle intensity over that time period. Then, the eigenvalues calculated for each acquisition channel over a given time period are selected and processed arithmetically according to a particular model to be used as data for the new channel. This processing method uses as input the EMG activity recorded by 2, 3, or 4 channels each time the hand performs a movement. The resulting sample of EMG data can be written as *S*. *S* is calculated as follows:

$$S=\left[\begin{array}{cccc} S_{11} & S_{12} & \cdots & S_{1n}\\ S_{21} & S_{22} & \cdots & \vdots \\ \vdots & \vdots & \cdots & \vdots \\ S_{m1} & S_{m2} & \cdots & S_{\mathrm{mn}} \end{array}\right],$$ (1)

where *n* is the number of EMG signal acquisition channels and *m* is the length of the sample. The data acquired within a 200-ms period are selected as samples in this paper. With a sampling frequency of 1 kHz, each sample contains 200 data points. Therefore, *m* = 200. Each element in *S* represents one sample data point. The collected samples are expressed as *S* = [*s*_1_, *s*_2_…*s_n_*], where each column represents all the data points collected from each channel.

Magnitude is an important feature describing the intensity of the EMG signal, which reflects the intensity of the muscle activity. The squared value of the sample data is calculated to obtain the intensity of activity of the corresponding muscle group during a given action as follows:

$$S^{2}=\left[\begin{array}{cccc} S_{11}^{2} & S_{12}^{2} & \cdots & S_{1n}^{2}\\ S_{21}^{2} & S_{22}^{2} & \cdots & \vdots \\ \vdots & \vdots & \cdots & \vdots \\ S_{m1}^{2} & S_{m2}^{2} & \cdots & S_{\mathrm{mn}}^{2} \end{array}\right].$$ (2)

To obtain information about the differences in muscle group activity across different hand gestures, the signal intensity difference between 2 muscles is selected as the added virtual channel in this paper. This information will be used to enrich the existing information about hand movements. The following processing steps are completed for the EMG samples acquired by each pair of channels.

For a given pair of columns of data in *S*, *S_i_* and *S_j_* represent the column vectors obtained from the EMG samples acquired by channel *i* and channel *j*, respectively, after the above processing; *S_k_* is the absolute value of the variance between the two. Since the magnitude of *S*^2^ can represent the intensity of muscle activity, the absolute value of the difference is used to represent the difference in the intensity of movement of 2 different muscles. *S_k_* is computed as follows:

$$S_{k}=|S_{i}^{2}-S_{j}^{2}|.$$ (3)

For all *S_k_*, *S_k_* and *S* are merged into the same sample, denoted as *S_new_*:

$$S_{\mathrm{new}}=\left[S,S_{k}\right]=\left[S_{1},S_{2}\ldots S_{n},S_{k}\right].$$ (4)

At this point, the number of columns of the signal of the sample is increased. The amount of EMG signal data is increased virtually without increasing the number of physical acquisition electrodes, providing more valid information for subsequent feature extraction and gesture classification. Each physical channel collects data that contains only information about a certain muscle group before processing. When the number of acquisition electrodes is high, more muscle movement information is provided for subsequent gesture recognition, therefore maintaining a high gesture recognition accuracy; however, when the number of acquisition channels is low, the amount of information provided by the acquisition electrodes is less, which leads to poor recognition. In contrast, after the EMG signal is processed to increase the number of dimensions virtually, the newly added information derived by fusing the information acquired by 2 channels uses the difference in the gesture intensity of different muscle groups to reflect the synergy and differences between different muscle groups during a certain gesture. The method of increasing the success rate of gesture recognition by simply adding more EMG acquisition electrodes tends to be ineffective after a certain number of acquisition electrodes are reached, and the above method provides a new solution to this problem.

#### EMG signal feature extraction and classification

Feature extraction from EMG signals is very important in the process of gesture recognition and analysis. Extracting the features of myoelectric signals can effectively improve the efficiency of data analysis. Feature vectors have the ability to represent data characteristics, so they can be used for the classification of different gestures. Based on previous research results, 3 features are selected: absolute standard deviation and root mean square of the time domain, and mean power frequency of the frequency domain.

Data must be normalized before being classified. For the various indicative gestures included here, a linear normalization method can be used due to the small amount of data and small data differentiation. The linear normalization function is shown as Eq. 5:

$$y=\frac{\left(x-x_{\min}\right)}{\left(x_{\max}-x_{\min}\right)},$$ (5)

then, the motion decision-making module uses previously trained classification algorithms to classify the virtual-dimensioned data from the myoelectric-signal acquisition channels into feature categories.

In terms of feature classification, the common methods for pattern classification are neural networks [17], support vector machines [18,19], Bayesian decision models [20,21], Markov models [22,23], and information fusion methods [24,25]. Among them, the back-propagation (BP) neural network classification method is widely used in many control fields involving nonlinear mapping because of its strong adaptive ability, generalization ability, and fault tolerance. Meanwhile, a large number of previous studies have shown that the BP neural network algorithm has good performance in motion intention recognition using human surface myoelectric-signal data [26–28]. Therefore, the proposed method uses the BP neural network algorithm to classify and identify the extracted features, and the model is designed based on the principle of the BP neural network algorithm. Finally, the control system of the prosthetic hand completes corresponding actions based on the classification results.

#### Gesture intent recognition strategy

**Fig. 2.**
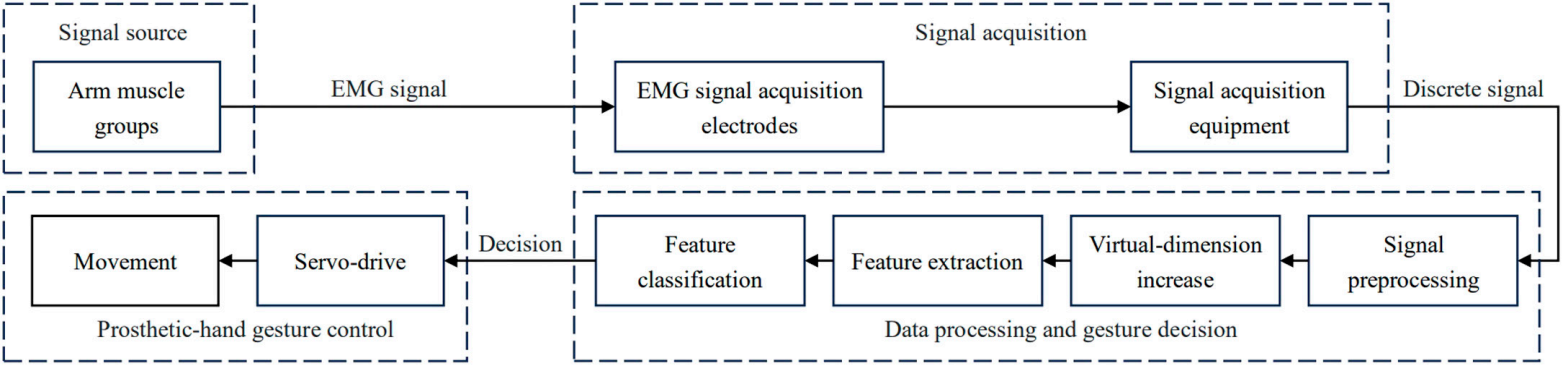
Control-flow diagram of prosthetic hands.

The control system of the intelligent myoelectric bionic hand used in this paper is divided into 4 main components: signal source module, signal acquisition module, data processing and gesture decision module, and prosthetic-hand gesture control module. Its basic control flow is shown in Fig. 2.

Firstly, the signal acquisition equipment obtains the discrete EMG signals from the arm muscle groups after which the signals are filtered and amplified and input into the data processing and gesture decision module, in which the multichannel signals are subjected to feature extraction and classification after increasing the virtual dimension. Finally, the classification result is input into the gesture control module of the prosthetic hand and controls the corresponding movements of the prosthetic hand by servo drive.

### Quality verification of gesture classification

In previous studies, classification effect has often been measured by the success rate of gesture recognition, with higher success rates of gesture recognition reflecting better classification effect. This validation approach is intuitive, but it does not reflect the inherent separability of the feature set, and the experimental results are susceptible to interference when the test sample is small, thus resulting in poor validation. To address this problem, this section will further process the feature vector data used for classification, reflect classification quality by evaluating classification performance, and quantify the separability of the feature set. We calculate the ratio of the interclass dispersion to intraclass dispersion of the feature data, which can more intuitively compare the advantages and disadvantages of different data processing and feature extraction methods and make preliminary predictions on the success rate of gesture recognition.

For the feature values extracted from discrete surface EMG signals, the average feature values for each acquisition channel can be expressed in the form of the following matrix:

$${\bar{X}}^{k}=\left[\begin{array}{c} {\bar{x}}_{1}^{k}\\ {\bar{x}}_{2}^{k}\\ \vdots \\ {\bar{x}}_{i}^{k} \end{array}\right],$$ (6)

among them:

$${\bar{x}}_{i}^{k}=\frac{1}{J}\sum_{j=1}^{J}x_{i,j}^{k}.$$ (7)

The standard deviation of the eigenvalues of each channel can be defined in the form of the following matrix:

$$S^{k}=\left[\begin{array}{c} s_{1}^{k}\\ s_{2}^{k}\\ \vdots \\ s_{i}^{k} \end{array}\right],$$ (8)

among them:

$$s_{i}^{k}=\sqrt{\frac{\sum_{j=1}^{J}{\left(x_{i,j}^{k}-{\bar{x}}_{i}^{k}\right)}^{2}}{J}},$$ (9)

*x* represents the eigenvalues normalized to the interval [0, 1], *i* represents the dimensionality of the feature vector extraction, *j* is the number of samples per action, and *k* is the action number. The statistical measure SFV can be expressed as:

$$\mathrm{SFV}=\frac{D}{c},$$ (10)

among them:

$$D=\frac{2}{K\left(K-1\right)}\sum_{p=1}^{K-1}\sum_{q=p+1}^{K}\sqrt{{\left({\bar{x}}_{1}^{p}-{\bar{x}}_{1}^{q}\right)}^{2}+{\left({\bar{x}}_{2}^{p}-{\bar{x}}_{2}^{q}\right)}^{2}+\cdots +{\left({\bar{x}}_{i}^{p}-{\bar{x}}_{i}^{q}\right)}^{2}},$$ (11)

where *p* and *q* represent action labels (1 = thumbs up, 2 = “V” gesture, 3 = “OK” gesture, 4 = clenched fist, 5 = open hand), and *i* represents the number of extracted eigenvalues. *D* is the distance between the coordinates of action *p* and action *q* clusters in the *n*-dimensional Euclidean space; larger values indicate more distinct differences between the eigenvalues of different actions, which in turn indicates that it is easier to distinguish them.

$$c=\frac{1}{\mathrm{IK}}\sum_{i=1}^{I}\sum_{k=1}^{K}s_{i}^{k},$$ (12)

*c* is the dispersion of the clusters of feature values of each action, and the smaller its value, the better the concentration of the feature values of the action and the easier it is to distinguish it from the feature values of other gestures.

From the above formula, SFV represents the ratio of interclass dispersion to intraclass dispersion of the feature dataset. Larger SFV values represent less differentiation of similar gesture features within the feature set and greater differentiation of different gestures, better differentiability of the feature set, and a higher success rate of its action intention recognition under the same conditions.

**Fig. 3.**
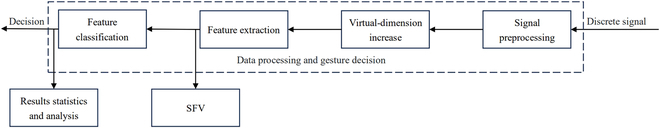
Quality verification process of the intention recognition strategy.

The validation of the classification quality by the SFV and the validation of the success rate of gesture recognition are performed at different stages in the control process of the prosthetic hands, as shown in Fig. 3, which shows the data processing and gesture decision module of the prosthetic-hands control strategy. The statistical recognition success rate is obtained after the feature set is classified. In contrast to the statistical action intention recognition success rate, the SFV saves the time required to verify the classification effect and is less affected by sample size, thus providing a new discriminatory basis for reflecting the classification quality of gesture intention recognition strategies.

## Experiments and Results

### Experimental platform

**Fig. 4.**
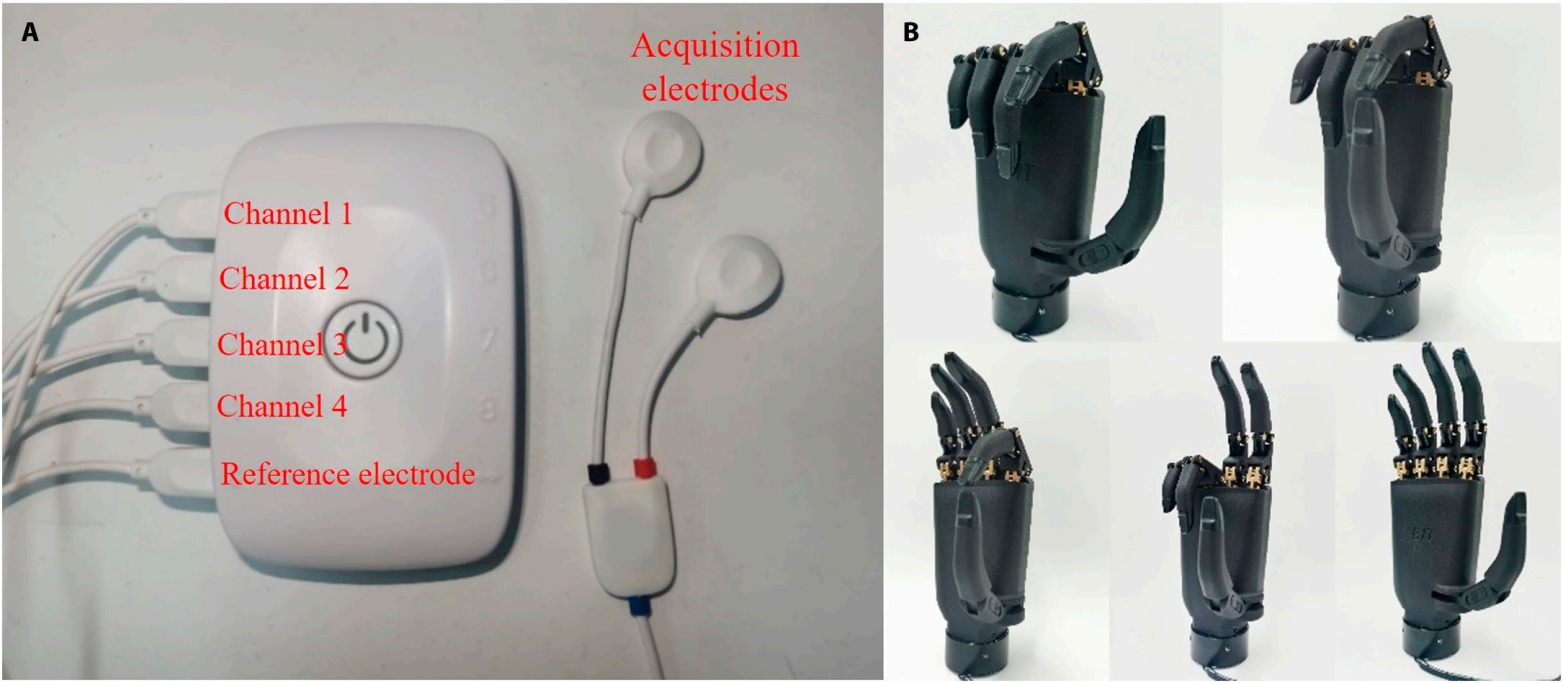
Experimental platform. (A) OpenSignals revolution acquisition platform. (B) Prosthetic hand imitating 5 gestures.

The data acquisition platform used in this experiment is OpenSignals revolution, which can visualize and record data in real time and perform preliminary signal processing. The physical equipment associated with the platform is shown in Fig. 4A. The intelligent bionic hand used in this experiment is the bionic hand described in Materials and Methods. The prosthetic hand can perform the 5 indicative gestures classified in this paper, and the 5 gestures made by the prosthetic hand are shown in Fig. 4B.

### Distribution of EMG electrodes

**Fig. 5.**
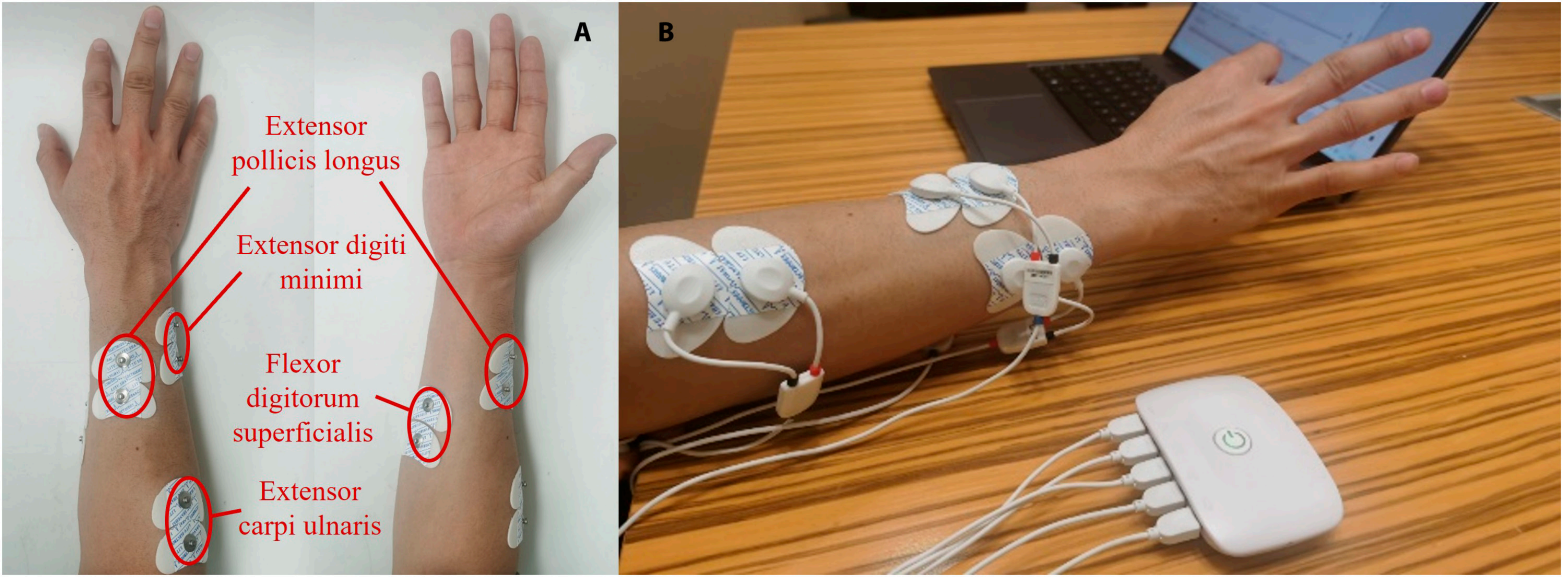
Electrode distribution and acquisition process. (A) Locations of the acquisition electrodes. (B) EMG signal acquisition.

The hand is able to perform different gestures under the control of different muscles or muscle groups in the arm. By analyzing the statistics of common gestures and the role of each muscle group in the arm, it is concluded that the motor information supporting hand movements is concentrated in the muscle groups in the arm. The thumb extensors are mainly used for extension of the thumb, and the state of the thumb extensors will change during “thumbs up”, “fist”, and “open hand” movements. The superficial thumb flexors and extensor carpi ulnaris are responsible for the flexion and extension of the other 4 fingers and their state will change during all movements except for the “thumbs up” movement; the extension of the little finger are mainly controlled by the extensor digiti minimi, which will change state during “OK”, “fist”, and “open hand” movements. At the same time, the strength of the muscle determines the strength of the EMG signal; the stronger the muscle is, the more accurate and rich the grip information from the corresponding EMG signal can be. After analyzing of function of the arm muscles and analysis of their contraction capacity was performed, for the 5 gestures identified in this paper, the following 4 muscle groups were ranked according to their number of participating movements and muscle strength: extensor carpi ulnaris, extensor pollicis longus, flexor digitorum superficialis, and extensor digiti minimi. EMG signals reflecting the activity of the 4 muscle groups were acquired, and the 4 groups of electrodes on the arm were arranged as shown in Fig. 5A. To facilitate the verification of the effect of virtual-dimension-increase-based gesture motion intention recognition and exclude the experimental errors caused by the atrophy or absence of muscle groups, subjects with healthy limbs were selected as the experimental subjects in this paper, and the data acquisition process is shown in Fig. 5B.

### Basic information for subjects

The soundness of the muscle groups that subjects need to use in this experiment, the state of the skin, and the adequacy of the surface EMG signal strength should be fully considered in the experimental process to ensure the validity of the experiment for the validation of the method proposed in this paper. Combining the above factors, and for the convenience of comparing the recognition effect of gestural motor intention and excluding the effect of muscle state on the virtual-dimension increase effect, we selected healthy young and middle-aged subjects as the experimental subjects. The 10 subjects in the experiments of this chapter were able-bodied and did not have any muscle-related diseases, and the specific information of the experimental subjects is shown in Table 2. Due to individual differences, the position of the electrodes applied to each subject is slightly different and needs to be adjusted according to the actual situation.

**Table 2.** Basic information of subjects

Number	Sex	Age	Arm for collecting signals	Muscle condition
1	Male	28	Right arm	Normal
2	Female	26	Left arm	Normal
3	Male	22	Right arm	Normal
4	Male	23	Right arm	Normal
5	Male	38	Left arm	Normal
6	Female	54	Right arm	Normal
7	Female	32	Right arm	Normal
8	Male	56	Left arm	Normal
9	Male	40	Left arm	Normal
10	Male	27	Left arm	Normal

### Experiment and analysis

Each subject was asked to repeat the 5 target actions: the thumbs-up gesture, the “V” gesture, the “OK” gesture, the fist and the open-hand gesture. A total of 150 samples were collected for each action. According to the research results of Khezri and Jahed [29], a length of 200 ms was considered appropriate for each sample. Here, the sampling frequency was set to 1,000 Hz; consequently, each sample contained 200 sampling points. For each action, there were 150 samples for each subject and 10 subjects in total, yielding 750 samples for each subject, and the ratio of the training set and the test set was set to 2:1. The training set was selected from the sample set of each subject, and the remaining samples formed the test set.

#### EMG signal virtual-dimension increase

To verify the improvement in the accuracy of gesture intention recognition due to the addition of virtual dimensions, the accuracy of gesture intention recognition was compared before and after the addition of virtual dimensions, and the results are reported in this section. The EMG signals were acquired, and gesture intention recognition was performed basing 2, 3, or 4 acquisition channels according to the method described in Materials and Methods. The preprocessed EMG signals were further processed to virtually increase their dimensionality, and the accuracy of recognition of 5 gestures before and after the virtual-dimension increase was recorded.

**Fig. 6.**
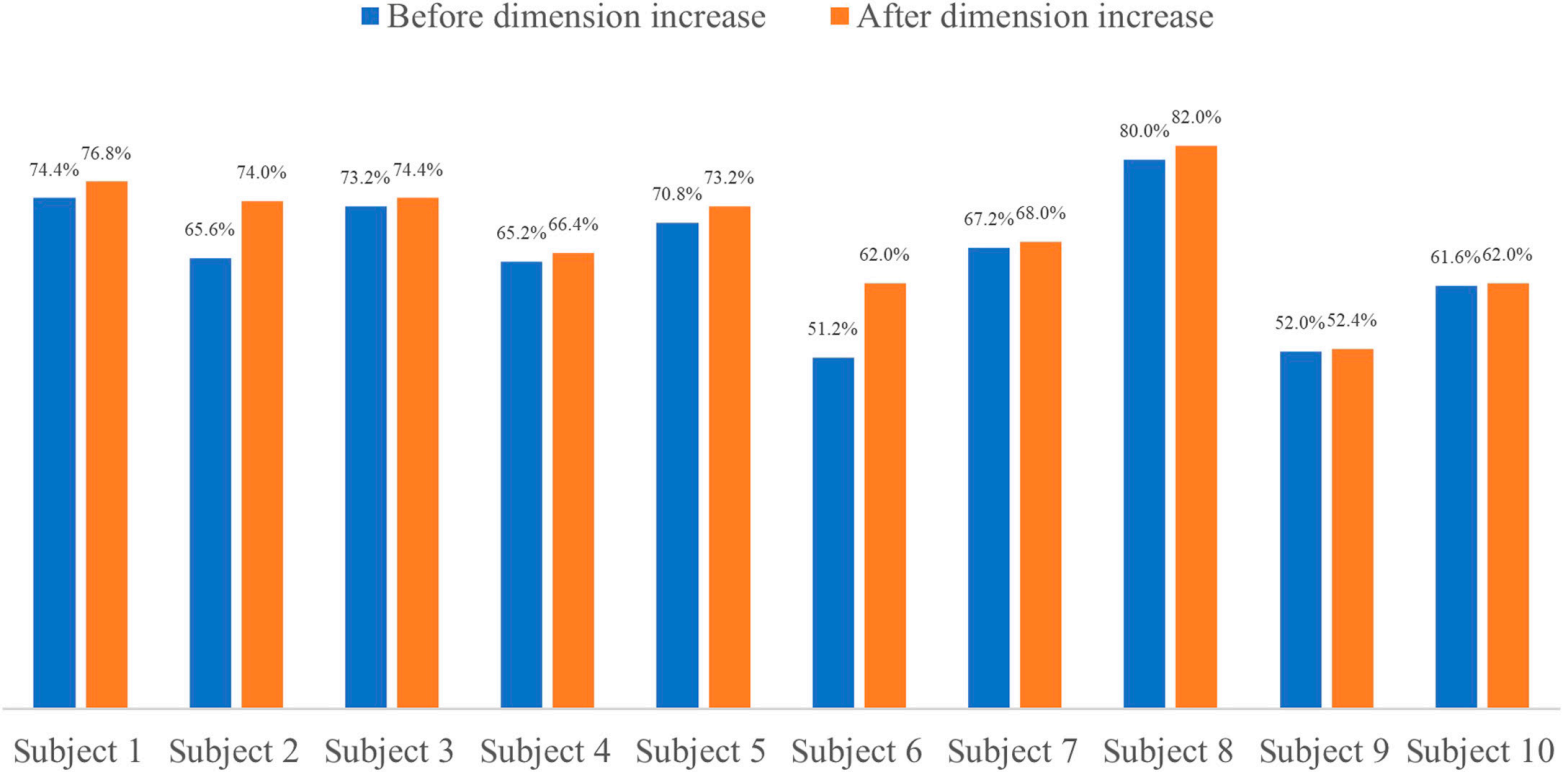
Accuracy of gesture recognition of 2-acquisition-channel signals before and after dimension increase.

First, dual acquisition electrodes were used for signal acquisition. Notably, the extensor carpi ulnaris and flexor digitorum superficialis were involved in more movements than the other muscle groups when the hand made the 5 movements required for the experiment. Also, the additional information that could be provided when performing interchannel information fusion was limited and had a small effect on the recognition rate. Therefore, the 2 selected acquisition channels correspond to the thumb extensors and extensor digiti minimi, respectively. The gesture recognition accuracy before and after the virtual-dimension increase is shown in Table 3, and the bar graph of the comparison is shown in Fig. 6.

**Table 3.** Accuracy of gesture recognition of 2-acquisition-channel signals before and after dimension increase.

Number	1	2	3	4	5
Recognition accuracy before dimension increase	74.4%	65.6%	73.2%	65.2%	70.8%
Recognition accuracy after dimension increase	76.8%	74%	74.4%	66.4%	73.2%
Number	6	7	8	9	10
Recognition accuracy before dimension increase	51.2%	67.2%	80%	52%	61.6%
Recognition accuracy after dimension increase	62%	68%	82%	52.4%	62%

**Fig. 7.**
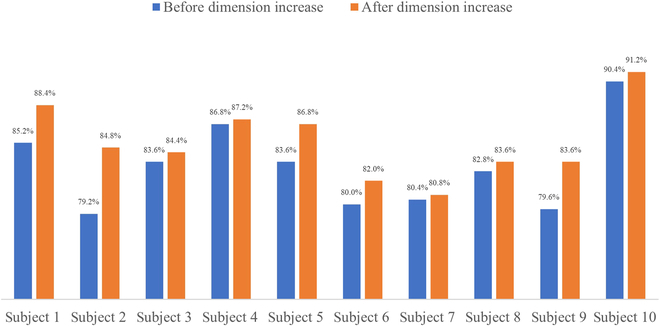
Accuracy of gesture recognition of 3-acquisition-channel signals before and after dimension increase.

After that, 3 acquisition electrodes were used for signal acquisition. Because the extensor carpi ulnaris is stronger and affects more of the 5 movements included in the experiment than the flexor digitorum superficialis, the additional information it can provide is limited, and it has a lower effect on the recognition rate. Therefore, the 3 acquisition channels correspond to the muscle groups of the extensor pollicis longus, flexor digitorum superficialis, and extensor digiti minimi. The gesture recognition accuracy rates before and after the virtual-dimension increase are shown in Table 4, and the bar graphs of the comparison are shown in Fig. 7.

**Table 4.** Accuracy of gesture recognition of 3-acquisition-channel signals before and after dimension increase

Number	1	2	3	4	5
Recognition accuracy before dimension increase	85.2%	79.2%	83.6%	86.8%	83.6%
Recognition accuracy after dimension increase	88.4%	84.8%	84.4%	87.2%	86.8%
Number	6	7	8	9	10
Recognition accuracy before dimension increase	80%	80.4%	82.8%	79.6%	90.4%
Recognition accuracy after dimension increase	82%	80.8%	83.6%	83.6%	91.2%

**Table 5.** Accuracy of gesture recognition of 4-acquisition-channel signals before and after dimension increase

Number	1	2	3	4	5
Recognition accuracy before dimension increase	86.8%	89.2%	85.6%	90.4%	88%
Recognition accuracy after dimension increase	90.8%	90.4%	86.8%	91.2%	89.2%
Number	6	7	8	9	10
Recognition accuracy before dimension increase	80.4%	87.2%	86.4%	94%	96.8%
Recognition accuracy after dimension increase	82.4%	89.6%	87.2%	95.2%	97.2%

**Fig. 8.**
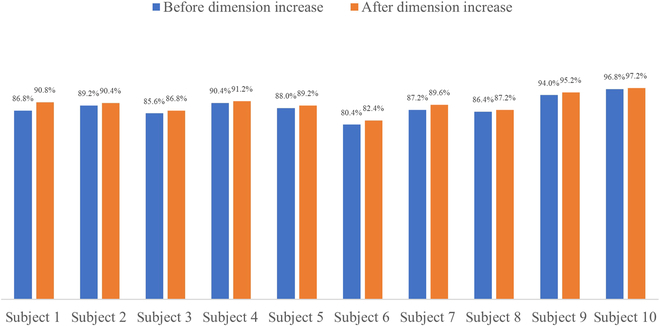
Accuracy of gesture recognition of 4-acquisition-channel signals before and after dimension increase.

Finally, all 4 acquisition electrodes were used for signal acquisition, and the channels corresponded to the extensor pollicis longus, flexor digitorum superficialis, extensor digiti minimi, and extensor carpi ulnaris. The gesture recognition accuracy before and after the virtual-dimension increase is shown in Table 5, and the bar graph of the comparison is shown in Fig. 8.

**Fig. 9.**
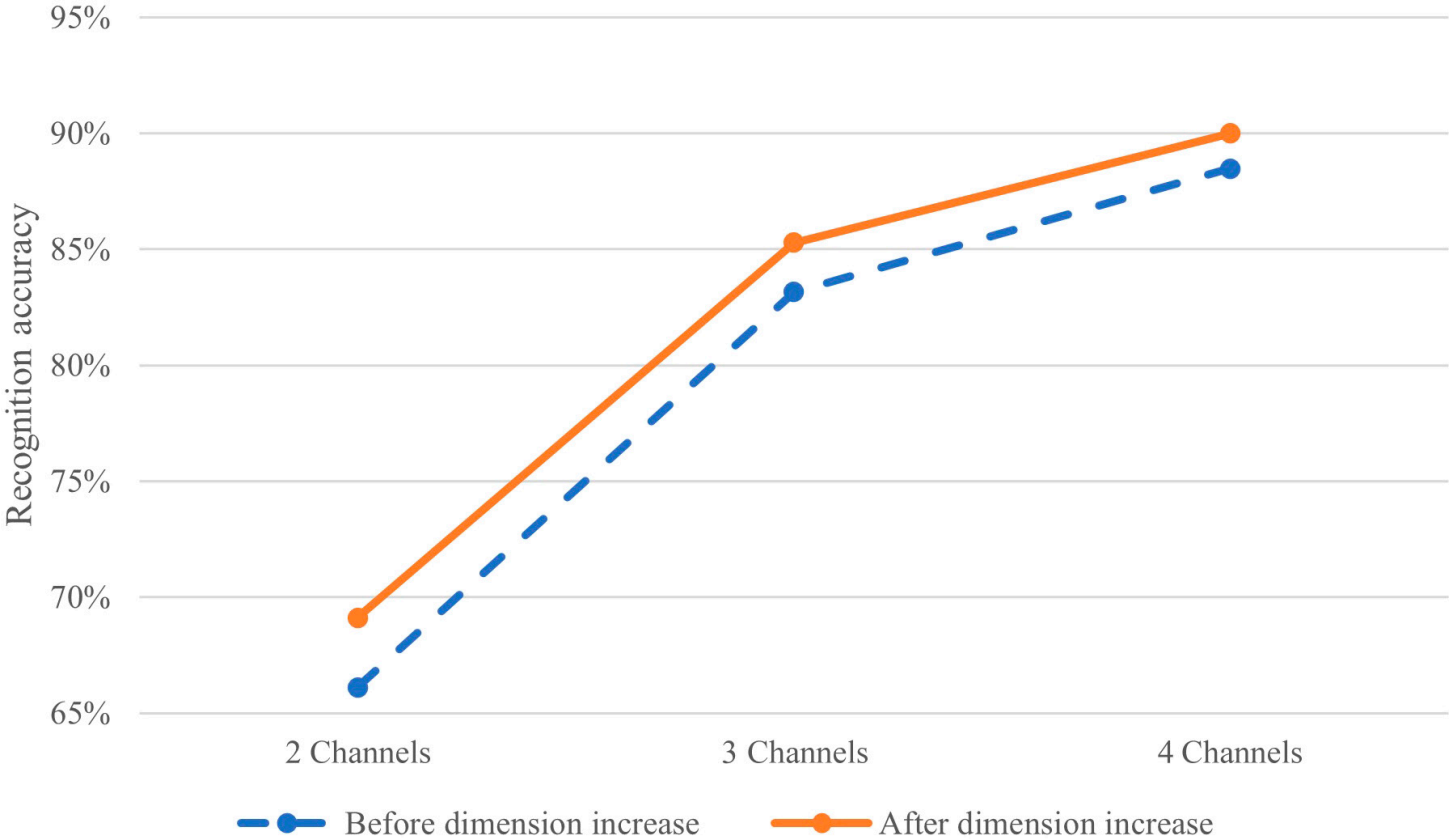
Change in gesture recognition accuracy before and after dimension increase with signals of different numbers of acquisition channels.

Line graphs of the average accuracy of classification before and after signal processing for each number of electrodes are shown in Fig. 9. The gesture recognition accuracy is higher after the virtual-dimension increase processing of the EMG signal than when using the unprocessed EMG signal. Also, the higher the number of EMG signal acquisition channels and the richer the EMG signal obtained, the higher the success rate of gesture recognition.

#### Separability verification

**Fig. 10.**
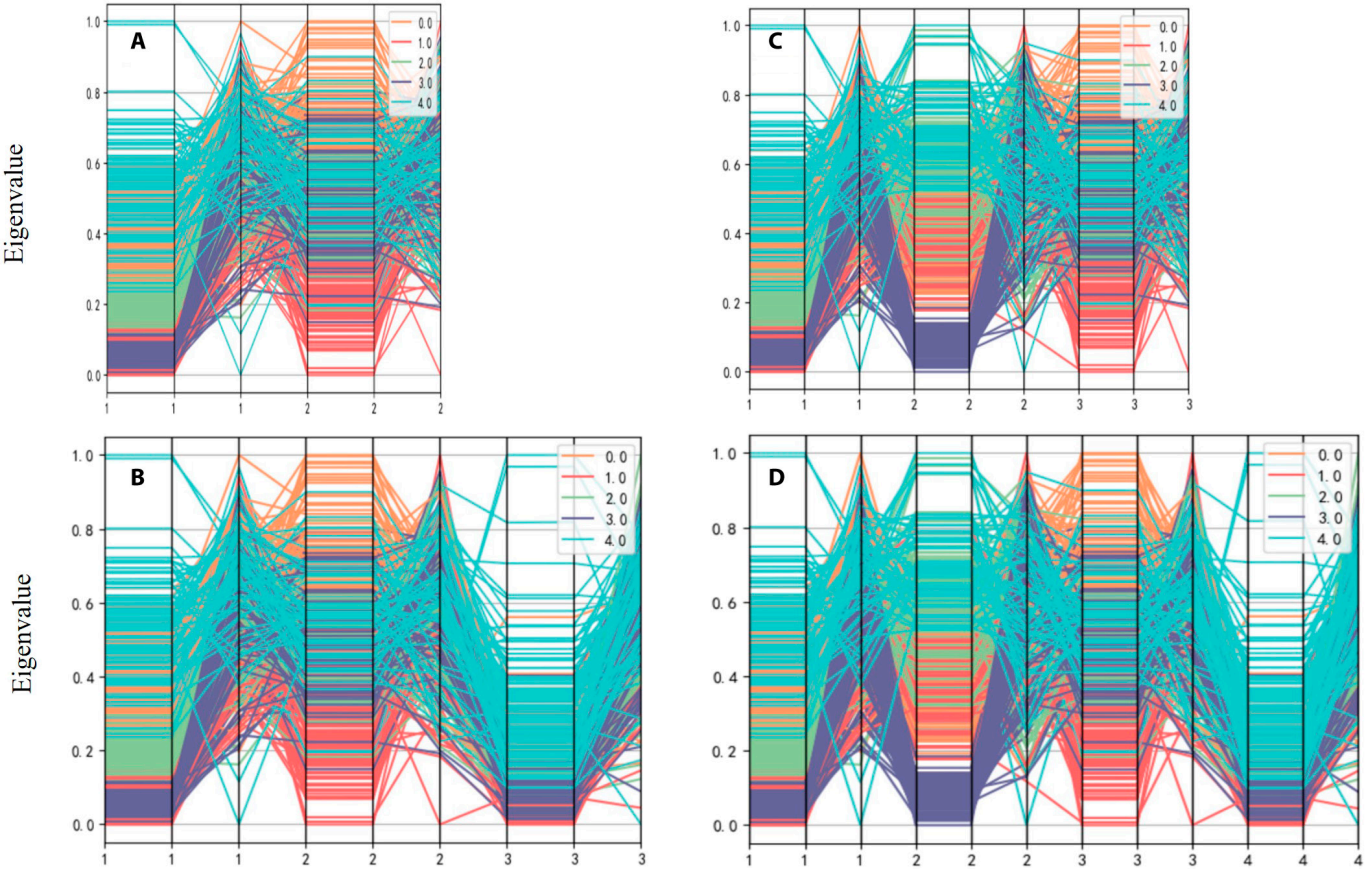
Feature sets of 2-acquisition-channel signals and 3-acquisition-channel signals before and after virtual-dimension increase. (A) Feature set of 2-acquisition-channel signals before virtual-dimension increase. (B) Feature set of 2-acquisition-channel signals after virtual-dimension increase. (C) Feature set of 3-acquisition-channel signals before virtual-dimension increase. (D) Feature set of 3-acquisition-channel signals after virtual-dimension increase.

To verify the effectiveness of SFV indicators in measuring gesture distinguishability and classification quality, this experiment calculates and compares the SFV values before and after the virtual-dimension increase of the feature set described in the previous experiments. These analyses reflect the connection between SFV values and the success rate of gesture intention recognition. First, the feature values extracted from the data from subjects are plotted in a parallel coordinate system, which allows for visualization of the changes in the differentiability of the 3 feature vector categories before and after the virtual-dimension increase for the 2 experiments, as shown in Fig. 10.

**Fig. 11.**
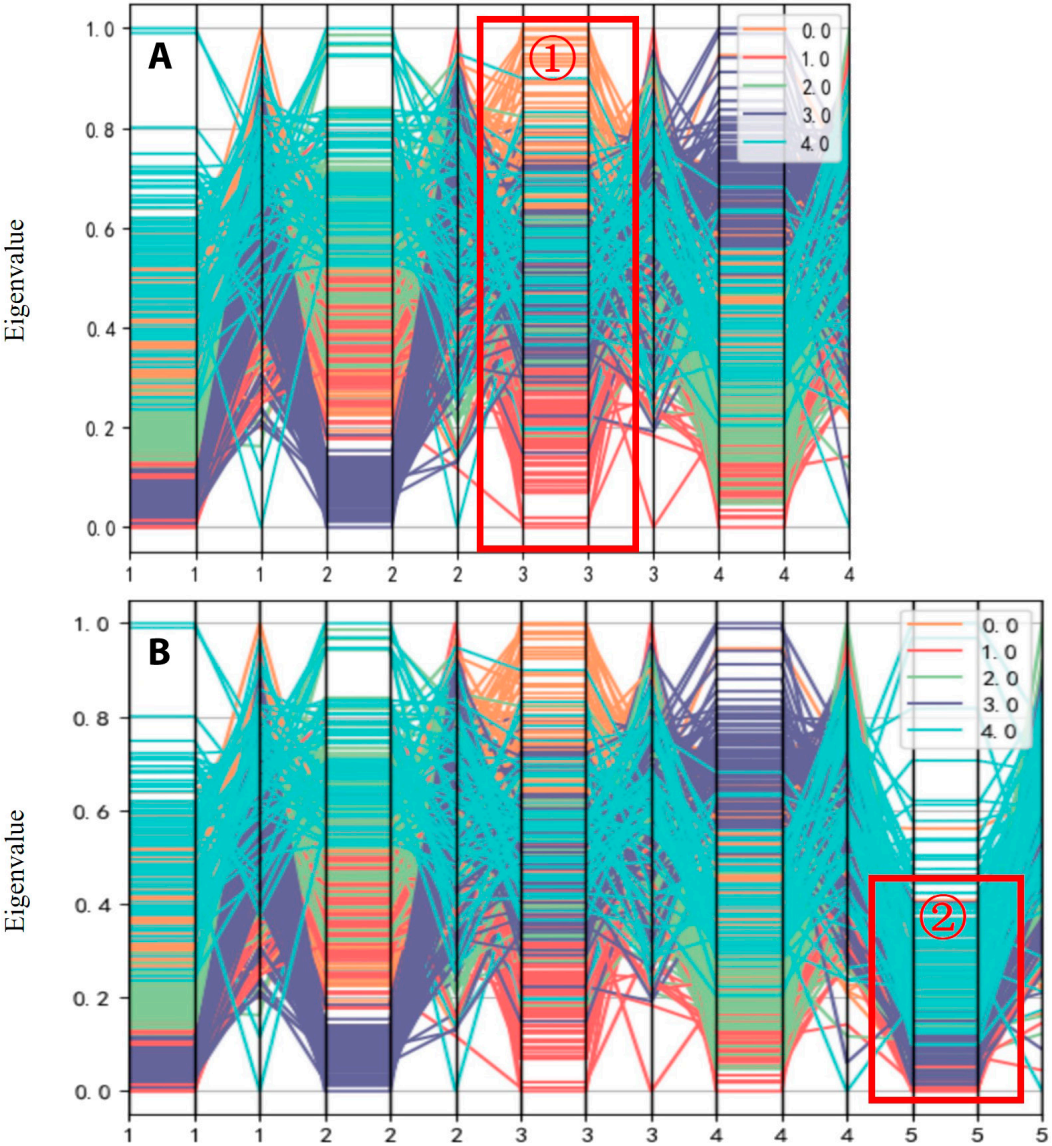
Feature sets of 4-acquisition-channel signals before and after virtual-dimension increase. (A) Feature set of 4-acquisition-channel signals before virtual-dimension increase. (B) Feature set of 4-acquisition-channel signals after virtual-dimension increase.

The horizontal coordinates in the figure represent the number of channels. Each channel has 3 eigenvalues, the vertical coordinates are the normalized eigenvalues, and the different colored line segments represent different gestures. In Figs. 10 and 11, the comparisons of the feature set of 5 gestures obtained before and after dimension increase of 2 to 4 channel signals are shown. Taking 4 channels as an example (Fig. 11A), the different color ranges representing the 5 gestures have high differentiation, indicating that the signals of different channels without virtual-dimension increase have obvious differentiability, reflecting the effectiveness of the acquisition location and the acquisition method. At the same time, Fig. 11B shows that the new signal with the virtual-dimension increase still has obvious distinguishability and can be complemented with the original signal channel information, which means that the new channel with the virtual-dimension increase reflects part of the implicit information of the original channel, and the virtual-dimension increase of the EMG signal can be used for the subsequent decision classification. The virtual-dimension addition process provides richer information about the gesture intention for subsequent decision classification.

The SFV values of the feature set before and after the virtual-dimension increase for subjects for different numbers of acquisition channels are shown in Tables 6 to 8.

Tables 6 to 8 show that the SFV increases after the virtual-dimension increase given the same number physical channels. According to the results of the theoretical analysis described in virtual-dimension increase, after virtual-dimension increase, additional information is reflected, which improves separability of feature sets and the recognition performance. This experimental conclusion verifies from the point of view of the variation in the separability of the feature set the validity of the principle behind virtual-dimension increase described in virtual-dimension increase. Furthermore, in comparing the trend of gesture intention recognition accuracy before and after virtual-dimension increase with the SFV, the larger the SFV is, the higher the recognition accuracy is, yielding a consistent conclusion with virtual-dimension increase experiment and reflecting the effectiveness of the SFV for measuring the differentiability of gestures and the gesture intention recognition effect.

**Table 6.** Comparison of SFV values of 2-acquisition-channel signals before and after virtual-dimension increase

Number	1	2	3	4	5
SFV (before dimension increase)	0.6102	0.6210	0.6464	0.8759	0.9531
SFV (after dimension increase)	0.8353	0.8810	0.8394	1.1034	1.1580
Number	6	7	8	9	10
SFV (before dimension increase)	0.8317	1.1525	0.6648	0.7227	0.8506
SFV (after dimension increase)	1.0625	1.4419	0.7847	0.8666	1.1510

**Table 7.** Comparison of SFV values of 3-acquisition-channel signals before and after virtual-dimension increase

Number	1	2	3	4	5
SFV (before dimension increase)	0.8045	0.8196	0.7327	1.0137	1.2654
SFV (after dimension increase)	0.9828	1.0487	0.8970	1.2162	1.4337
Number	6	7	8	9	10
SFV (before dimension increase)	1.1395	1.4230	0.8230	0.9017	1.3163
SFV (after dimension increase)	1.3401	1.6745	0.9260	1.0267	1.5710

**Table 8.** Comparison of SFV values of 4-acquisition-channel signals before and after virtual-dimension increase

Number	1	2	3	4	5
SFV (before dimension increase)	0.9524	1.0603	0.8951	1.1057	1.2799
SFV (after dimension increase)	1.1122	1.2291	1.0365	1.2794	1.4471
Number	6	7	8	9	10
SFV (before dimension increase)	1.3619	1.6265	0.9100	0.9824	1.5539
SFV (after dimension increase)	1.5368	1.8422	0.9972	1.0884	1.7745

Experiment 2: In the recognition of gestures based on EMG signal control, the recognition success rate is usually used to reflect the classification effect. However, when the number of samples is insufficient, the results may have large deviation and cannot effectively reflect the classification effect it should have. Therefore, in this section, 60 to 150 samples are randomly selected from the sample set to form a subsample set, and the ratio of the number of samples in the training set and the test set in the subsample set is 2:1. The gesture recognition accuracy and SFV values under 2, 3, and 4 acquisition channels are recorded, and the data results of some sample sets are listed in Tables 9 to 11.

**Table 9.** Comparison of recognition accuracy of 2-acquisition-channel signals and SFV values for small samples

Number of samples	60	90	120	150
Recognition accuracy	40%	63.3%	65%	70%
SFV	0.7674	0.8211	0.9808	0.8180

**Table 10.** Comparison of recognition accuracy of 3-acquisition-channel signals and SFV values for small samples

Number of samples	60	90	120	150
Recognition accuracy	90%	83.3%	87.5%	84%
SFV	0.9066	0.9397	1.2068	1.0932

**Table 11.** Comparison of recognition accuracy of 4-acquisition-channel signals and SFV values for small samples

Number of samples	60	90	120	150
Recognition accuracy	85%	86.7%	82.5%	88%
SFV	1.055	0.9947	1.2484	1.1393

**Fig. 12.**
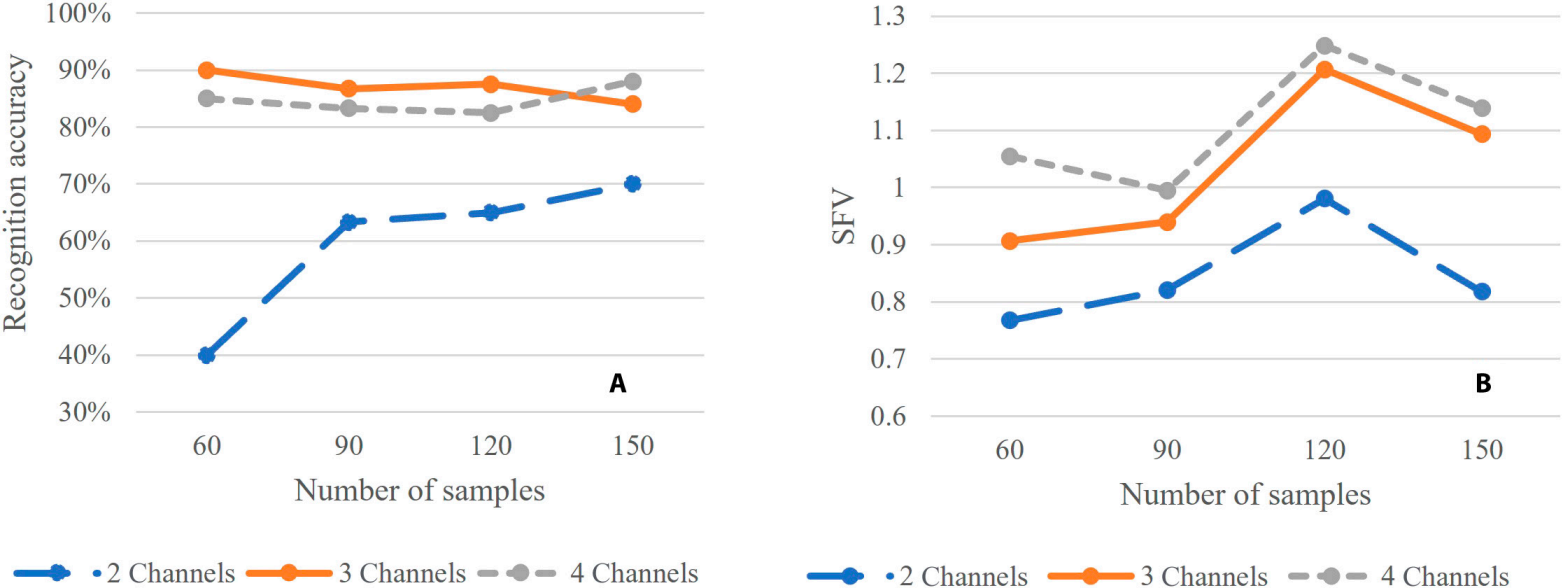
The accuracy of action recognition and SFV values for small sample sets. (A) Variation in the action recognition accuracy of 2-, 3-, and 4-acquisition-channel signals for small samples. (B) Variation in the SFV values of 2-, 3-, and 4-acquisition-channel signals for small samples.

Figure 12A shows the result records of the accuracy of action recognition with 2, 3, and 4 acquisition channels in the subsample set. The recognition accuracy does not improve strictly with the increase in the number of channels, which indicates that the use of accuracy to reflect classification effects in the case of small samples is prone to bias, making the results neither universal nor representative. Figure 12B shows the results recorded for the SFV values of 2, 3, and 4 acquisition channels in the subsample set. The SFV values increase with the increase in the number of acquisition channels, which indicates that the SFV values can still reflect the separability of the sample set in the small sample case.

## Discussion

In this paper, we propose a method for increasing the dimensions of EMG signals virtually to obtain richer gesture movement intention information and improve the recognition accuracy of different hand movements without adding additional EMG signal acquisition electrodes. Compared with the traditional EMG signal processing method, this method can extract more effective movement intention information from the existing EMG signals through the correspondence between movements and muscle groups without adding any additional hardware. By comparing the action recognition accuracy of the EMG signals based on 2, 3, and 4 acquisition channels before and after the virtual-dimension increase, we verified that the gesture recognition accuracy improves after the virtual-dimension increase processing. The method provides a solution to the problem of EMG information saturating after a certain number of electrodes.

Meanwhile, for the gesture classification quality verification part, a classification quality verification method based on SFV is proposed in this paper. SFV represents the ratio of interclass dispersion to intraclass dispersion in the feature dataset. The larger SFV values indicate smaller differences among similar gestures and the greater differences between different gestures in the feature set. By comparing the SFV values of EMG signals obtained by 2, 3, and 4 acquisition channels before and after the increase of virtual dimension in the experiment, we showed that after virtual-dimension increase, additional information is added and the feature set separability is improved and the recognition performance is further improved. Meanwhile, by comparing the accuracy of gesture intention recognition before and after the increase of virtual dimension with the SFV value, we showed that the larger the SFV value is, the higher the recognition accuracy, which reflects the effectiveness of the SFV index in measuring the differentiability of gestures and the gesture intention recognition effect.

Finally, a random small-sample experiment was conducted. The results showed that in the case of small samples, the recognition accuracy does not necessarily increase with the increase in the number of signal acquisition channels, while the SFV value becomes larger with the increase in the number of acquisition channels. It can be concluded that when using the accuracy rate to reflect classification effects, there is a risk of bias, making the results neither universal nor representative. However, the SFV value can still reflect the separability in the sample set. Thus, compared with the statistical action intention recognition success rate, the SFV value can effectively predict the classification effect and with less interference from the number of samples.

According to the above experiments, the virtual-dimension increase of EMG signal can obtain richer gesture motion intention information by virtually increasing the EMG signal channels, so as to improve the recognition accuracy of different gestures without increasing the hardware equipment, and this method provides a solution to the problem of saturating the EMG information provided after increasing the number of electrodes. In the gesture classification quality verification session, compared with the classification effect measured by statistical motion intention recognition success rate, SFV is faster and the results are more representative, and the effectiveness of the virtual-dimension increase strategy is verified from the perspective of feature set separability change through the experiment; in the case of small sample capacity, the classification effect is prone to bias by statistical recognition success rate, while SFV can still reflect the separability of the sample set more accurately.

## Data Availability

The data used to support the findings of this study are included within the article.

## References

[1] Laffranchi M, Boccardo N, Traverso S, Lombardi L, Canepa M, Lince A, Semprini M, Saglia JA, Naceri A, Sacchetti R, et al. The hannes hand prosthesis replicates the key biological properties of the human hand. Sci Robot. 2020;5(46):eabb0467. 32967990 10.1126/scirobotics.abb0467

[2] Gu G, Zhang N, Xu H, Lin S, Yu Y, Chai G, Ge L, Yang H, Shao Q, Sheng X, et al. A soft neuroprosthetic hand providing simultaneous myoelectric control and tactile feedback. Nat Biomed Eng. 2021;7(4):1–10. 10.1038/s41551-021-00767-0 34400808

[3] Leone F, Gentile C, Ciancio AL, Gruppioni E, Davalli A, Sacchetti R, Guglielmelli E, Zollo L. Simultaneous semg classification of hand/wrist gestures and forces. Front Neurorobot. 2019;13:42. 31275131 10.3389/fnbot.2019.00042 PMC6593108

[4] Jiang S, Lv B, Guo W, Zhang C, Wang H, Sheng X, Shull PB. Feasibility of wrist-worn, real-time hand, and surface gesture recognition via semg and imu sensing. IEEE Trans Industr Inform. 2017;14(8):3376–3385.

[5] Bo L, Banghua Y, Shouwei G, Yan L, Zhuang H, Wang W. Hand gesture recognition using semg signals based on cnn. Paper presented at: 2021 40th Chinese Control Conference (CCC); 2021 Jul 26–28; Shanghai, China.

[6] Trigno® Maize System - Delsys. [accessed 12 Feb 2023] http://delsys.com/trigno-maize

[7] Hardware - OT Bioelettronica. [accessed 12 Feb 2023] https://www.otbioelettronica.it/prodotti/hardware

[8] SAGA 32+_64+_128+ - High Density Amplifier - TMSi. [accessed 12 Feb 2023] https://www.tmsi.com/products/saga-32-64

[9] He J, Sheng X, Zhu X, Jiang C, Jiang N. Spatial information enhances myoelectric control performance with only two channels. IEEE Trans Industr Inform. 2019;15(2):1226–1233.

[10] Krasoulis A, Kyranou I, Erden MS, Nazarpour K, Vijayakumar S. Improved prosthetic hand control with concurrent use of myoelectric and inertial measurements. J Neuroeng Rehabil. 2017;14:1–14. 28697795 10.1186/s12984-017-0284-4 PMC5505040

[11] Naik GR, Al-Timemy AH, Nguyen HT. Transradial amputee gesture classification using an optimal number of semg sensors: An approach using Ica clustering. IEEE Trans Neural Syst Rehabil Eng. 2016;24(8):837–846. 26394431 10.1109/TNSRE.2015.2478138

[12] Ameur S, Khalifa AB, Bouhlel MS. Chronological pattern indexing: An efficient feature extraction method for hand gesture recognition with leap motion. J Vis Commun Image Represent. 2020;70: Article 102842.

[13] Li Y, He Z, Ye X, He Z, Han K. Spatial temporal graph convolutional networks for skeleton-based dynamic hand gesture recognition. EURASIP J Image Video Process. 2019;2019(1):1–7.

[14] He J, Zhu X. Combining improved gray-level co-occurrence matrix with high density grid for myoelectric control robustness to electrode shift. IEEE Trans Neural Syst Rehabil Eng. 2017;25(9):1539–1548. 28026779 10.1109/TNSRE.2016.2644264

[15] Kaas JH. *Evolution of nervous systems*. 2nd ed. Oxford (UK): Academic Press, 2017.

[16] Alba DM, Moyà-Solà S, Köhler M. Morphological affinities of the australopithecus afarensis hand on the basis of manual proportions and relative thumb length. J Hum Evol. 2003;44(2):225–254. 12662944 10.1016/s0047-2484(02)00207-5

[17] Yamanoi Y, Togo S, Jiang Y, Yokoi H. Learning data correction for myoelectric hand based on “survival of the fittest”. Cyborg Bionic Syst. 2021;2021:9875814. 36285147 10.34133/2021/9875814 PMC9494700

[18] Wang B, Wang C, Wang L, Xie N, Wei W. Recognition of semg hand actions based on cloud adaptive quantum chaos ions motion algorithm optimized svm. J Mech Med Biol. 2019;19(06):1950047.

[19] Wen T, Zhang Z, Qiu M, Zeng M, Luo W. A two-dimensional matrix image based feature extraction method for classification of semg: A comparative analysis based on svm, knn and rbf-nn. J Xray Sci Technol. 2017;25(2):287–300. 28269818 10.3233/XST-17260

[20] Tello RM, Bastos-Filho T, Costa RM, Frizera-Neto A, Arjunan S, Kumar D. Towards semg classification based on bayesian and k-nn to control a prosthetic hand. Paper presented at: 2013 ISSNIP Biosignals and Biorobotics Conference: Biosignals and Robotics for Better and Safer Living (BRC); 2013 Feb 18–20; Rio de Janeiro, Brazil.

[21] Caza-Szoka M, Massicotte D, Nougarou F. Naive bayesian learning for small training samples: application on chronic low back pain diagnostic with semg sensors. Paper presented at: 2015 IEEE International Instrumentation and Measurement Technology Conference (I2MTC) Proceedings; 2015 May 11–14; Pisa, Italy.

[22] Rossi M, Benatti S, Farella E, Benini L. Hybrid emg classifier based on hmm and svm for hand gesture recognition in prosthetics. Paper presented at: 2015 IEEE International Conference on Industrial Technology (ICIT); 2015 Mar 17–19; Seville, Spain.

[23] Cheng J, Chen X, Liu A, Peng H. A novel phonology-and radical-coded chinese sign language recognition framework using accelerometer and surface electromyography sensors. Sensors. 2015;15(9):23303–23324. 26389907 10.3390/s150923303 PMC4610461

[24] Zhong T, Li D, Wang J, Xu J, An Z, Zhu Y. Fusion learning for semg recognition of multiple upper-limb rehabilitation movements. Sensors. 2021;21(16):5385. 34450825 10.3390/s21165385 PMC8398355

[25] Zhang Y, Chen S, Cao W, Guo P, Gao D, Wang M, Zhou J, Wang T. Mffnet: Multi-dimensional feature fusion network based on attention mechanism for semg analysis to detect muscle fatigue. Expert Syst Appl. 2021;185: Article 115639.

[26] Lei Z. An upper limb movement estimation from electromyography by using bp neural network. Biomed Sig Process Control. 2019;49:434–439.

[27] Zhang F, Li P, Hou Z-G, Lu Z, Chen Y, Li Q, Tan M. Semg-based continuous estimation of joint angles of human legs by using bp neural network. Neurocomputing. 2012;78(1):139–148.

[28] Motion estimation of elbow joint from semg using continuous wavelet transform and back propagation neural networks. Biomed Sig Process Control. 2021;68: Article 102657.

[29] Khezri M, Jahed M. A neuro–fuzzy inference system for semg-based identification of hand motion commands. IEEE Trans Ind Electron. 2010;58(5):1952–1960.

